# Prospective Analysis of Safety and Efficacy of Tenofovir Alafenamide Fumarate (TAF) in European Real-World Patients with Chronic Hepatitis B: A Single-Centre Real-Word Cohort Study

**DOI:** 10.3390/pathogens13090820

**Published:** 2024-09-23

**Authors:** Balazs Fülöp, Janett Fischer, Magdalena Hahn, Albrecht Böhlig, Madlen Matz-Soja, Thomas Berg, Florian van Bömmel

**Affiliations:** 1 Division of Hepatology, Department of Medicine II, Leipzig University Medical Center, 04103 Leipzig, Germany; balazs.fueloep@ksbl.ch (B.F.); janett.fischer@medizin.uni-leipzig.de (J.F.); magdalena.hahn@medizin.uni-leipzig.de (M.H.); madlen.matz-soja@medizin.uni-leipzig.de (M.M.-S.); thomas.berg@medizin.uni-leipzig.de (T.B.); 2 Klinik Gastroenterologie und Hepatology, Kantonsspital Baselland, 4410 Liestal, Switzerland; 3 Department of Internal Medicine, Community Hospital Delitzsch, 34208 Delitzsch, Germany; albrecht.boehlig@medizin.uni-leipzig.de

**Keywords:** tenofovir alafenamide, real-world cohort, chronic Hepatitis B

## Abstract

**Background:** Tenofovir alafenamide (TAF) is a novel prodrug of tenofovir for the treatment of chronic hepatitis B (CHB) that has shown a favourable renal safety profile while offering suppression of HBV DNA similar to tenofovir disoproxil fumarate (TDF). We aimed to study changes in markers of HBV replication and renal function in a real-world setting in European patients. **Methods:** In our prospective single-arm, non-interventional observational study, HBeAg-positive and HBeAg-negative patients with chronic HBV mono-infection receiving TAF as their first or following line treatment were enrolled. HBV DNA, HBsAg, markers of bone metabolism, and renal function were determined at baseline and every consecutive 3 months. **Results:** A total of 50 patients (70% male) were included. The mean duration of TAF treatment was 18 (3–36) months. In 20 patients with detectable HBV DNA at baseline, median serum levels of HBV DNA log_10_ changed from 2.33 (0.766–6.47) to 1.04 IU/mL at the end of observation and became undetectable in 11 patients. Median HBsAg log_10_ decreased from 3.37 (0.88–5.10) to 2.39 (1.52–4.19) IU/mL. During the entire observation period, the renal function parameters remained stable in patients with normal renal function and even in those with renal dysfunction. Mild adverse events were reported by 14 patients (28%). **Conclusions:** TAF was a safe and effective treatment, also in patients with decreased renal function.

## 1. Introduction

Chronic hepatitis B (CHB) is a significant global health challenge, affecting millions of individuals worldwide. The primary goals of CHB treatment are to suppress hepatitis B virus (HBV) replication, prevent liver disease progression, and reduce the risk of complications such as cirrhosis and hepatocellular carcinoma (HCC) [1,2]. Tenofovir disoproxil fumarate (TDF) has been a cornerstone in the treatment of CHB; however, its long-term use can be associated with renal toxicity and bone density reduction [3,4,5,6,7].

Tenofovir alafenamide (TAF) is a novel prodrug of tenofovir that delivers the active drug more efficiently to hepatocytes, allowing for lower dosing and reduced systemic exposure. This results in improved renal and bone safety profile compared to TDF [8,9]. Clinical trials have demonstrated the efficacy and safety of TAF in suppressing HBV DNA, but real-world data are essential to confirm these findings in a broader patient population [10].

This study aimed to evaluate the safety and efficacy of TAF in a single-centre real-world cohort of patients with CHB, with a specific focus on changes in HBV replication markers, renal function, and markers of bone density.

## 2. Methods

### 2.1. Study Design and Participants

This prospective single-arm, non-interventional observational study included HBeAg-positive and HBeAg-negative patients aged over 18 years with chronic HBV monoinfection. The use of TAF was based on the individual decision of the treating physician. Patients were either naïve to TAF treatment or had received it as a follow-up treatment. This study was approved by the Ethics Committees of Medical Research of the University of Leipzig in accordance with the Declaration of Helsinki from 1975 (revision 2013) and the International Conference on Harmonization/Committee for Proprietary Medicinal Products “Good Clinical Practice” guidelines. Written informed consent was obtained from all participants. Exclusion criteria included co-infection with other hepatitis viruses or human immunodeficiency virus (HIV), and any severe uncontrolled comorbidity.

### 2.2. Data Collection

Data were collected at baseline and every three months thereafter, including demographic information, HBV DNA levels, HBsAg levels, markers of bone metabolism, and renal function indicators. The primary endpoints were changes in HBV DNA and HBsAg levels, while secondary endpoints included renal function (creatinine clearance (CrCl), serum creatinine), bone metabolism markers (parathormone, beta-2-microglobulin, urinary albumin), and adverse events. The patients were divided into the following subgroups based on their renal function as reflected in CrCl: severe (CrCl < 30 mL/min), moderate (CrCl 30–60 mL/min), or mild renal impairment (60–90 mL/min) and normal renal function (CrCl > 90 mL/min).

### 2.3. Statistical Analysis

Statistical analyses of epidemiological associations were performed using SPSS software (SPSS Inc., version 24.0, Chicago, IL, USA) and GraphPad Prism (GraphPad Prism Inc., version 10.0.2, Boston, MA, USA). Descriptive statistics were used to summarize patient demographics and baseline characteristics. Changes in HBV DNA, HBsAg, renal function, and bone metabolism markers were analyzed using paired Wilcoxon tests. All tests were two-sided and *p*-values less than 0.05 were considered statistically significant. Adverse events were reported as frequencies and percentages.

## 3. Results

### 3.1. Patient Characteristics

A total of 50 patients (70% male, median 56 (range 20–83) years, median BMI 27.0 (range 17.2–51.01)) who presented at Leipzig University Medical centre from 2017 to 2021 were prospectively included in the study. The majority were Caucasian (74%), and 40 (80%) had HBeAg-negative CHB. Five patients had compensated liver cirrhosis, and one patient had HCC. At baseline, severe impaired renal function (CrCl < 30 mL/min) was present in 4% of patients, moderate (CrCl 30–60 mL/min) in 26% of patients, and mild (60–90 mL/min) renal impairment in 34% of patients. The mean duration of TAF treatment was 12 (range 3–36) months (Table 1). A total of 8 patients (16%) received TAF as the first line of treatment, and 42 patients (84%) as the following line treatment.

### 3.2. Virological Response

**Figure 1:**
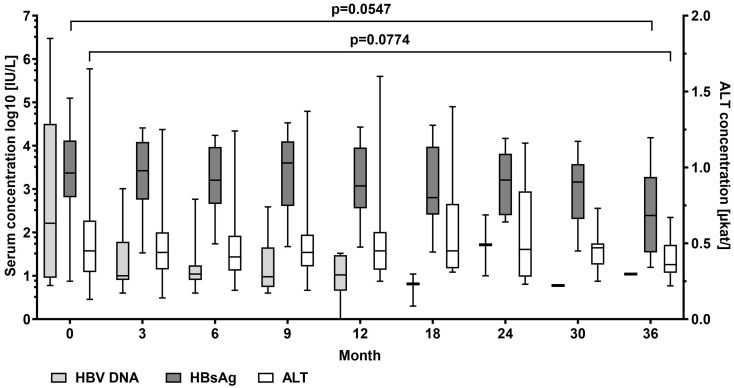
Hepatitis B virus (HBV) DNA, HBV surface antigen (HBsAg), and ALT alanine aminotransferase serum levels at baseline and at month 3, 9, 12, 18, 24, 30, and 36 during tenofovir alafenamide (TAF) treatment. Wilcoxon test was used to compared baseline values and values at month 36.

In patients with detectable HBV DNA at baseline (*n* = 20), mean serum levels of HBV DNA log_10_ decreased from median 2.19 (0.76–6.47) to 1.04 IU/mL at the end of the observation period. HBV DNA log_10_ became undetectable in 11 patients. Mean HBsAg log_10_ levels decreased from median 3.37 (0.88–5.10) to 2.39 (1.20–4.19) IU/mL, *p* = 0.055. No patient experienced HBsAg loss. Median alanine aminotransferase (ALT) serum levels decreased from 0.45 (0.13–1.65) µkat/L at the start of TAF treatment to 0.36 (0.22–0.67) µkat/L, *p* = 0.077) at the end of observation (Figure 1).

### 3.3. Fibrosis Indicators

**Figure 2:**
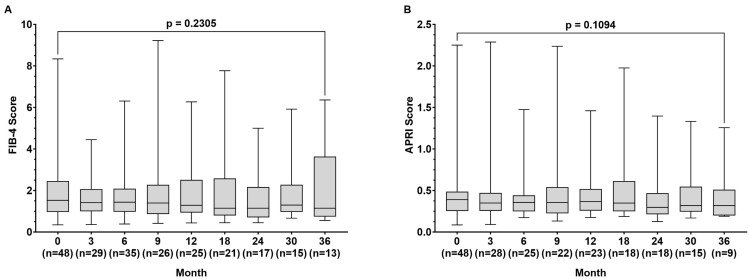
Fibrosis-4 (FIB-4) score (**A**) and aspartate aminotransferase to platelet ratio index (APRI) score (**B**) at baseline and at month 3, 9, 12, 18, 24, 30, and 36 during tenofovir alafenamide (TAF) treatment. Wilcoxon test was used to compared baseline values and values at month 36.

During the observation time, the FIB-4 index (1.53 (0.35–78.34) vs. 1.15 (0.56–5.80) *p* = 0.231) remained stable, while the APRI score improved slightly from baseline to month 36, but without being statistically significant (0.391 (0.086–2.250) vs. (0.320 (0.190–1.257) *p* = 0.109) (Figure 2A,B).

### 3.4. Renal Function and Bone Metabolism

**Figure 3:**
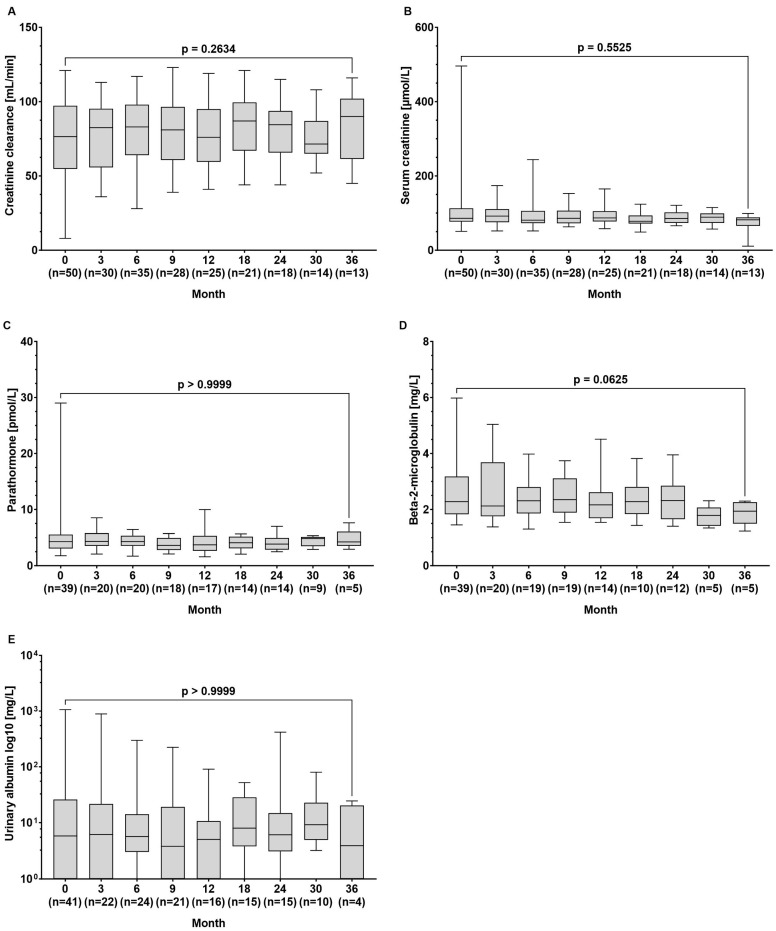
Serum concentrations of the renal function parameters of (**A**) creatinine clearance, (**B**) creatinine, (**C**) parathormone, (**D**) beta-2-microglobulin, and (**E**) urinary albumin at baseline and during tenofovir alafenamide (TAF) treatment. Wilcoxon test was used to compared baseline values and values at month 36.

The median creatinine clearance (77 (8–121) vs. 90 (45–116) µmol/L, *p* = 0.263) and serum creatinine levels (86 (51–496) vs. 82 (21–99) min/L, *p* = 0.552) remained stable throughout the observation period. Additionally, there were no significant changes in the median concentrations of parathormone (4.30 (1.79–29.0) vs. 4.25 (2.94–7.61) pmol/L, *p* > 0.999), beta-2-microglobulin (2.28 (1.45–5.98) vs. 1.94 (1.23–2.30) mg/L, *p* = 0.063), and urinary albumin (5.8 (0–1062) vs. 3.9 (0–24.4) mg/L, *p* > 0.999) between baseline and the end of observation (Figure 3A–E).

**Figure 4:**
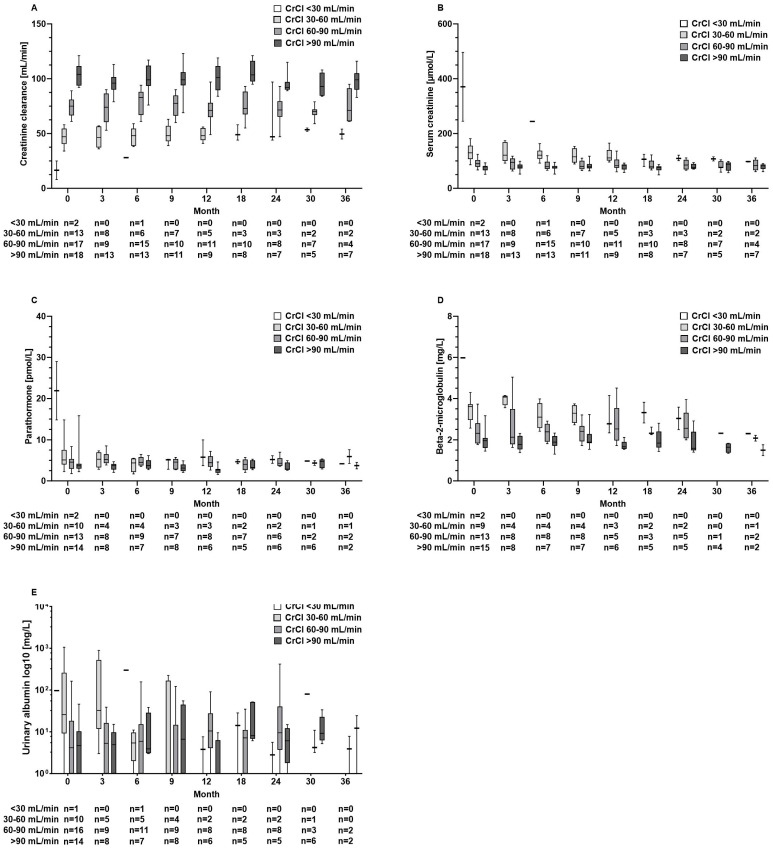
Serum concentrations of renal function parameters of (**A**) creatinine clearance, (**B**) creatinine, (**C**) parathormone, (**D**) beta-2-microglobulin, and (**E**) urinary albumin at baseline and during tenofovir alafenamide fumarate (TAF) treatment according to the renal function groups with severe (CrCl < 30 mL/min), moderate (CrCl 30–60 mL/min), or mild renal impairment (60–90 mL/min) and normal function (CrCl > 90 mL/min).

Also, in patients with abnormal kidney function results at baseline, kidney function parameters and parathormone levels did not further deteriorate during TAF treatment (Figure 4A–E). Furthermore, these aforementioned parameters remained unaltered in the TDF pre-treated subgroup (*n* = 26) (Figure S1A–E).

### 3.5. Adverse Events

Adverse event grade 1 or 2 that were potentially related to TAF treatment was reported by 14 patients (28%). These included exanthema (*n* = 2), headache (*n* = 2), fatigue (*n* = 3), abdominal pain (*n* = 3), arthralgia (*n* = 1), non-exertional dyspnoea and perspiration (*n* = 1), dizziness (*n* = 1), and increases in creatine kinase (CK), aspartate aminotransferase (AST), and lactate dehydrogenase (LDH) levels (*n* = 1). Three patients discontinued TAF due to adverse events. No adverse event grade 3 or 4 was reported (Table 2). One patient died during the study period without any connection to liver disease or TAF intake. 

## 4. Discussion

Our real-world study confirms that TAF is a safe and effective treatment option for patients with CHB, including those with impaired renal function at the start of treatment. The significant reduction in HBV DNA levels and the stability of renal function markers highlight the clinical benefits of TAF. Our findings are consistent with previous clinical trials and provide further evidence supporting the use of TAF in routine clinical practise [6,7,8,9,10].

### 4.1. Antiviral Efficacy of TAF

HBV DNA levels showed a continuous decrease during TAF treatment and were undetectable in nine patients and below 2000 IU/mL in four patients after 12 months of treatment. No patient showed an increase in HBV DNA levels. HBsAg levels remained positive during the observation period, showing a slight tendency to decrease (Figure 1). This was in accordance with a study by Sano et al [11]. They recently assessed the efficacy of switching from adefovir dipivoxil and TDF therapy to TAF for the treatment of CHB. The researchers observed a reduction in serum HBsAg levels and an improvement in other markers after six months of treatment [11]. However, no patient lost HBsAg during the observation period, which is in line with other observations on long-term NA treatment in predominantly HBeAg-negative patient populations [7,8,12,13,14,15].

Furthermore, TAF was associated with durable ALT normalization. The results of our study are consistent with those of a recent meta-analysis, which reported significant improvements in mean ALT levels and ALT normalization following a switch to TAF [7,8,16].

### 4.2. Renal Safety

Renal safety is a critical concern in the long-term management of CHB, particularly given the nephrotoxic potential of some antiviral therapies. TDF, although effective, is associated with declines in renal function and an increased risk of renal tubular dysfunction. In contrast, TAF has shown a more favourable renal safety profile, even in HBV/HIV-co-infected patients [7,8,17,18]. Accordingly, in our patient cohort, we did not observe a decrease in GFR or an increase in creatinine levels over time. Moreover, beta-2-microglobulin levels, which are considered a marker for tubular injury, remained unchanged (Figure 3). Testing for albumin in the urine as part of the basic diagnostics for suspected nephropathy did not reveal loss of kidney function. Also, TAF did not cause decreases in kidney function parameters in 33 patients with decreased renal function at baseline (Figure 4B). Our results confirm the favourable renal safety of TAF in a real-world situation across a broad spectrum of biomarkers [7,8].

Several studies have highlighted the renal safety of TAF [8,9,19]. For instance, Agarwal et al. (2018) demonstrated that patients treated with TAF had stable renal function over a 96-week period compared to those treated with TDF, who experienced significant declines in creatinine clearance [8]. Similarly, Buti et al. (2016) reported that TAF is associated with smaller increases in serum creatinine and smaller declines in the estimated glomerular filtration rate [6]. Additionally, a meta-analysis of data from numerous studies revealed that patients treated with TAF exhibited only a small decline in renal function during the course of treatment when compared to controls [20].

### 4.3. Serum Markers of Bone Density

Parathormone levels, which may be altered in secondary hyperparathyroidism as a result of hypocalcaemia and hyperphosphataemia (e.g., in the context of chronic renal insufficiency or liver cirrhosis), remained stable in our population (Figure 3). These findings are in line with results of prospective studies revealing that bone density decreased significantly less during 48 and 96 weeks of treatment with TAF than with TDF [7,8,20]. Our real-world data support the use of TAF in patients with risk of bone density decrease, which may affect, for example, patients with liver cirrhosis or patients of advanced age.

During the observation time, the mean results for the FIB-4 index remained stable in our patient population, while the APRI score slightly improved but did not reach statistical significance (*p* = 0.109) (Figure 2). These observations are in line with another real-life study on TAF treatment in 270 patients in which changes in FIB-4 were not detected after 48 weeks of treatment [21]. In contrast, another study on 53 patients treated with TAF over 144 weeks, including 8 patients with liver cirrhosis, showed improvement in FIB-4 results, suggesting that a longer follow-up might have resulted in a stronger decrease in FIB-4 levels in our patients [22]. The mild impact of TAF treatment on liver fibrosis in our population is also likely based on the relatively small number of patients with liver cirrhosis (Table 1). 

A limitation of our study is the relatively small sample size. During the time of the data collection, the use of TAF was reimbursed by the health insurance companies in Germany, so no specific indication or special assumption of costs by the insurance company was necessary. Today, benefits of treatment with TAF are offset by the limited availability and reimbursement of costs, as well as the lack of cost-effectiveness in many regions with regard to the current price level [23,24]. It is questionable whether a change in pricing and reimbursement policy or the introduction of generic medication could boost the use of TAF in the future.

Our study provides real-world evidence supporting the safety and efficacy of TAF in European patients with chronic hepatitis B, including those with impaired renal function. Based on the available data, the EASL guideline recommends that TAF (or entecavir if possible) in patients with osteopenia/osteoporosis and renal insufficiency in preference to TDF [2]. The results of our study may help us to understand the clinical benefit of TAF for future therapies due to the favourable renal safety profile under TAF treatment, and it adds clinical real-world evidence to the potential role of TAF in the management of CHB [25,26]. Further real-world studies are warranted to further validate our findings and to provide the basis for the development of a broader use of TAF.

## Figures and Tables

**Table 1:** Baseline patient characteristics of the study cohort.

Parameter	Number/Value
Male (%)	35 (70%)
Age (years)	56 (20–83)
Ethnicity (%)	
Caucasian	37 (74%)
Asian	8 (16%)
Middle East	4 (8%)
African	1 (2%)
BMI (kg/cm^2^) *	27.0 (17.2–51.1)
Diabetes (%)	12 (24%)
Hyperlipoproteinemia (%)	9 (18%)
Liver cirrhosis (%)	5 (10%)
HCC (%)	1 (2%)
Detectable HBV DNA (%)	20 (40%)
HBV DNA concentration (log_10_ IU/mL) *	2.33 (0.76–6.47)
HBeAg-positive (%)	10 (20%)
HBsAg concentration (log_10_ IU/mL) *	3.38 (0.88–5.10)
Previous HBV treatment (%)	41 (82%)
Tenofovir (%)	26 (63%)
Entecavir (%)	13 (32%)
Lamivudine (%)	1 (2%)
Adefovir (%)	1 (2%)
Duration of TAF treatment (month) *	18 (3–36)
ALT (µkat/mL) *	0.45 (0.13–1.65)
AST (µkat/mL) *	0.49 (0.27–1.32)
AP (µkat/mL) *	1.35 (0.59–3.23)
GGT (µkat/mL) *	0.41 (0.14–7.05)
Albumin (g/L) *	45.9 (36.0–51.5)
Bilirubin (µmol/L) *	8.5 (2.9–27.9)
Hemoglobin (mmol/L) *	8.9 (6.2–10.9)
Platelets (×10^9^/L) *	213 (57–489)
Leucocytes (×10^9^/L) *	6.4 (1.4–17.9)
FIB-4 score *	1.53 (0.35–8.34)
APRI score *	0.391 (0.086–2.250)
Creatinine (µmol/L) *	86 (51–496)
Creatinine clearance (mL/min) *	77 (8–121)
CrCl < 30 mL/min (%)	2 (4%)
CrCl 30–60 mL/min (%)	13 (26%)
CrCl 60–90 mL/min (%)	17 (34%)
CrCl > 90 mL/min (%)	18 (36%)
Beta-2-microglobulin (mg/L) *	2.28 (1.45–5.98)
Urinary albumin (mg/L) *	5.8 (0–1062)
Cholesterol (mmol/L) *	4.20 (2.96–7.40)
HDL (mmol/L) *	1.27 (0.80–2.01)
LDL (mmol/L) *	2.79 (1.49–5.83)
Triglyceride (mmol/L) *	1.23 (0.65–4.49)
Bonespecific alkaline phosphatase (µkat/mL) *	18.5 (6.3–44.6)
Parathormon (pmol/L) *	4.30 (1.79–29.0)
25-Hydroxycholecalciferol (ng/mL) *	22.5 (6.6–50.7)

* median (range), ALT: alanine aminotransferase, AP: alkaline phosphatase, APRI: aspartate aminotransferase to platelet ratio index, AST: aspartate aminotransferase, BMI: body mass index, CrCl: creatinine clearance, FIB-4: fibrosis-4, GGT: gamma-glutamyl transpeptitase, HBeAg: HBV envelope antigen, HBsAg: HBV surface antigen, HBV: hepatitis B virus, HDL: high-density lipoprotein, HCC: hepatocellular carcinoma, IU: international unit, LDL: low-density lipoprotein.

**Table 2:** Adverse events related to tenofovir alafenamide (TAF) treatment.

Adverse Event	Number
Mild adverse events (%)	14 (28%)
Exanthema	2 (14%)
Headache	2 (14%)
Fatigue	3 (21%)
Abdominal pain	3 (21%)
Arthalgia	1 (7%)
Non-exertional dyspnea/perspiration	1 (7%)
Dizzines	1 (7%)
CK, AST, and LDH increase	1 (7%)
Severe adverse events	0

AST: aspartate aminotransferase, CK: creatine kinase, LDH: lactate dehydrogenase.

## Data Availability

The data presented in this study are available upon request from the corresponding author.

## Supplementary Materials

The following supporting information can be downloaded at: https://www.mdpi.com/article/10.3390/pathogens13090820/s1, Figure S1: Serum concentrations of the renal function parameters (A) creatinine clearance, (B) creatinine, (C) parathormone, (D) beta-2-microglobulin and (E) urinary albumin at baseline and during Tenofovir alafenamide (TAF) treatment in the subgroup of patients pre-treated with Tenofovir disoproxil fumarate (TDF).

## Abbreviations

ALT: alanine aminotransferase, AP: alkaline phosphatase, APRI: aspartate aminotransferase-to-platelet ratio index, AST: aspartate aminotransferase, CHB: chronic hepatitis B, CrCl: creatinine clearance, DNA: deoxyribonucleic acid, FIB-4: fibrosis-4, GGT: gamma-glutamyl transpeptitase, HBeAg: HBV envelope antigen, HBsAg: surface antigen, HBV: hepatitis B virus, HCC: hepatocellular carcinoma, HDL: high-density lipoprotein, HIV: human immunodeficiency virus, IU: international unit, LDL: low-density lipoprotein, TAF: tenofovir alafenamide, TDF: tenofovir disoproxil fumarate.
