# Supplementary material for: Prospective Analysis of Safety and Efficacy of Tenofovir Alafenamide Fumarate (TAF) in European Real-World Patients with Chronic Hepatitis B: A Single-Centre Real-Word Cohort Study
Source: Pathogens. 2024 Sep 23;13(9):820. doi: 10.3390/pathogens13090820 (PMC11434708; doi:10.3390/pathogens13090820)
Supplement: Supplementary file 1 [file pathogens-13-00820-s001.zip › pathogens-3108662-supplementary.pdf]

Article

Prospective Analysis of Safety and Efficacy of Tenofovir Alafenamide Fumarate (TAF) in European Real-World Patients with Chronic Hepatitis B: A Single-Centre Real-Word Cohort Study

Balazs Fülöp [1,2], Janett Fischer [1], Magdalena Hahn [1], Albrecht Böhlig [3], Madlen Matz-Soja [1], Thomas Berg [1] and Florian van Bömmel [1,*]

[1] Division of Hepatology, Department of Medicine II, Leipzig University Medical Center, 04103 Leipzig, Germany; balazs.fueloep@ksbl.ch (B.F.); janett.fischer@medizin.uni-leipzig.de (J.F.); magdalena.hahn@medizin.uni-leipzig.de (M.H.); madlen.matz-soja@medizin.uni-leipzig.de (M.M.-S.); thomas.berg@medizin.uni-leipzig.de (T.B.)
[2] Kantonsspital Baselland, Klinik Gastroenterologie und Hepatology, 4410 Liestal, Switzerland
[3] Department of Internal Medicine, Community Hospital Delitzsch, 34208 Delitzsch, Germany;
albrecht.boehlig@medizin.uni-leipzig.de
* **Correspondence: florian.vanboemmel@medizin.uni-leipzig.de**

Table of contents

Supplementary Results

Figures S1 2

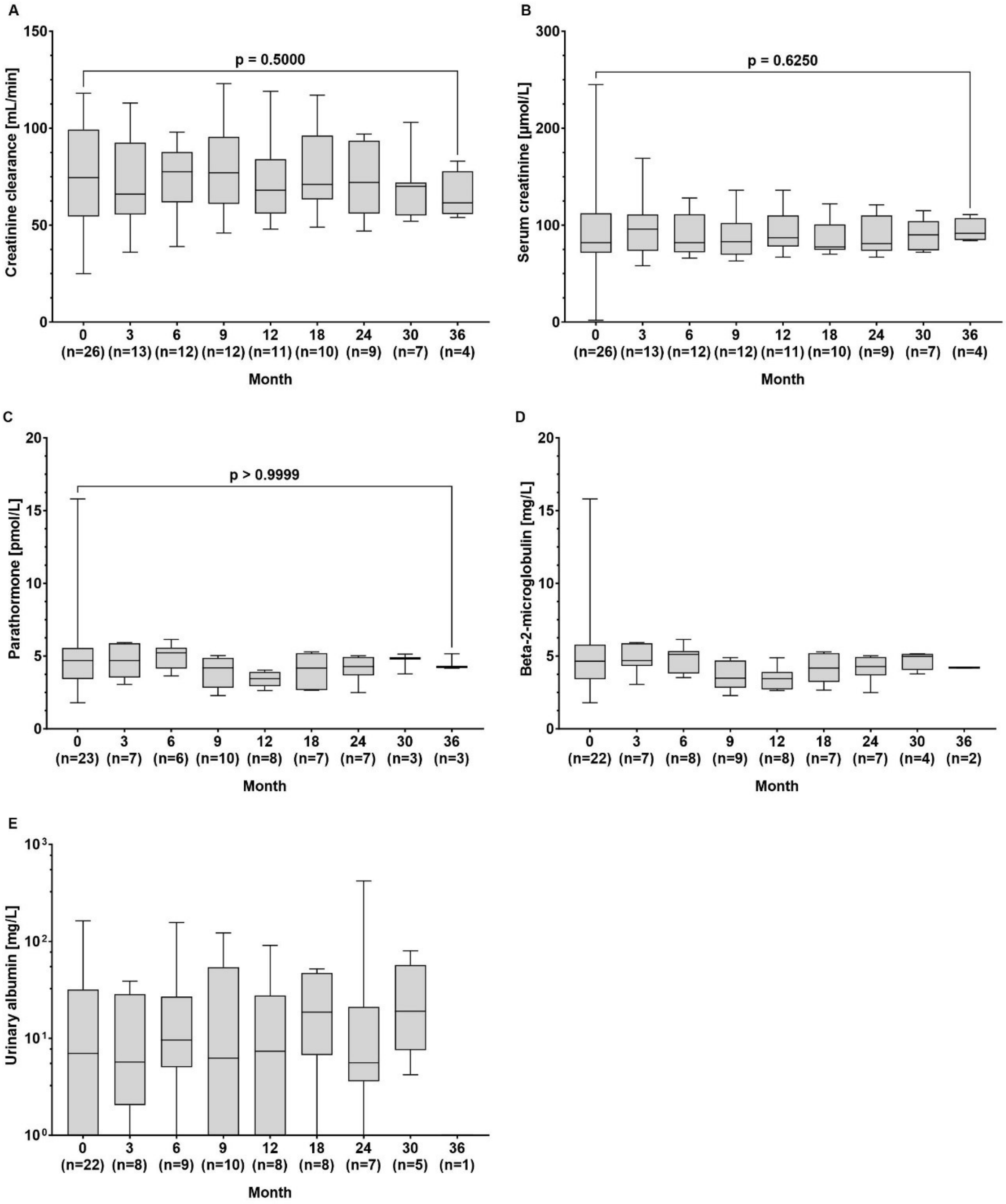

Figure S1: Serum concentrations of the renal function parameters (**A**) creatinine clearance, (**B**) creatinine, (**C**) parathormone, (**D**) beta-2-microglobulin and (**E**) urinary albumin at baseline and during Tenofovir alafenamide (TAF) treatment in the subgroup of patients pre-treated with Tenofovir disoproxil fumarate (TDF).
